# Surgical site infections after abdominal surgeries: a prospective multicentre study in 53 Nigerian hospitals

**DOI:** 10.1136/bmjgh-2025-021423

**Published:** 2026-06-04

**Authors:** Nigerian Surgical Research Collaborative, Adewale Adisa

**Keywords:** Africa South of the Sahara, Indices of health and disease and standardisation of rates, Surgery

## Abstract

**Introduction:**

Surgical site infection (SSI) remains a significant challenge in surgical care, particularly in low- and middle-income countries like Nigeria. Limited access to reliable, up-to-date data has hindered efforts to reduce this burden. This study provides a snapshot audit of SSI incidence and influencing factors in Nigeria, the largest country in sub-Saharan Africa.

**Methods:**

We conducted a multicentre prospective cohort study using a collaborative network of surgeons across 53 hospitals in Nigeria between June and August 2024, enrolling 2466 patients undergoing abdominal operations with incisions of at least 5 cm in adults or 3 cm in children. Caesarean sections were excluded. SSI was defined based on Centres for Disease Control and Prevention criteria and monitored at discharge and on postoperative day 30. Predefined risk factors assessed included patient characteristics, surgical factors and perioperative practices. Associations were examined using a multilevel logistic regression model with hospital as a random intercept to account for clustering.

**Results:**

Of the 2466 recruited patients, 2240 were included in the final analysis. Over half (56.4%) underwent emergency surgery. Open procedures were performed in 98.3% (2202) of cases, with general anaesthesia administered to 77.8% (1741) of patients. The WHO Surgical Safety Checklist was used in 69.9% (1564) of cases. Overall, 26.4% (591) of patients developed SSI within 30 days postsurgery. Predictors of SSI include preoperative haematocrit (packed cell volume), smoking status, HIV status and emergency surgery. In a multivariable analysis, American Society of Anaesthesiologists (ASA) grades 3–5: OR 1.91 (1.48 to 2.45, p<0.001), contaminated/dirty contamination: OR 5.48 (3.33 to 9.01, p<0.001) and open midline approach: OR 1.41 (1.06 to 1.88, p=0.020) were significantly associated with higher odds of SSI.

**Conclusion:**

Abdominal surgeries in Nigeria are associated with a high SSI rate, which contributes to adverse postoperative outcomes in patients. Identifying modifiable risk factors could help inform national policies aimed at SSI prevention.

**WHAT IS ALREADY KNOWN ON THIS TOPIC**

- Reducing the burden of surgical site infections (SSI) in Nigeria and other sub-Saharan African countries has been challenging due to the lack of reliable local data. Previous retrospective and single-centre studies have not provided sufficient evidence to guide effective interventions and planning.

**WHAT THIS STUDY ADD**

- This snapshot audit identified a 26.4% rate of SSI following abdominal surgeries across 53 hospitals in 32 states and the Federal Capital Territory of Nigeria. Surgeries performed on ASA 3–5 patients were associated with high SSI rates. SSI significantly increased the risk of readmission, reoperation, prolonged hospital stay and mortality.

**HOW THIS STUDY MIGHT AFFECT RESEARCH, PRACTICE OR POLICY**

- The modifiable risk factors identified are opportunities for targeted institutional and national intervention strategies to improve surgical outcomes and reduce the unacceptably high SSI rates.

## Introduction

 Surgical site infection (SSI) is the most common complication following abdominal operations worldwide, posing a significant burden on patients, healthcare facilities and health systems.^1 2^ A large international, multicentre study found that SSI rates following abdominal surgeries were disproportionately higher in low- and middle-income countries (LMICs) compared with high-Human Development Index (HDI) countries, with reported incidences of 23.2%, 14.0% and 9.2%, respectively.^3^ These infections contribute to increased morbidity, mortality and healthcare costs, particularly in resource-limited settings.^4 5^

As Africa’s most populous country, Nigeria faces a substantial share of this burden. While various interventions to reduce SSI have been recommended, especially in low-resource environments,^6^ their implementation requires robust, reliable data from nationwide, multicentre observational studies. Currently, most reports on SSI incidence in Nigeria rely on retrospective, single-centre studies with limited patient data.^7^,^9^ The lack of active SSI surveillance in these studies also weakened the findings of a recent meta-analysis.^10^ As a result, previous studies cannot be reliably used to inform national planning and policy decisions on SSI prevention.

The primary objective of this study was to determine the incidence of postoperative SSI within 30 days of abdominal surgery in both adults and children across Nigeria. The secondary objectives were to evaluate 30-day postoperative mortality rates and identify the factors that influence SSI occurrence and patient outcomes.

## Methodology

The Association of Surgeons of Nigeria, at its 2023 Annual Meeting in Lagos, Nigeria, set up a Steering Committee that worked in collaboration with the Nigeria Hub of the UK National Institute of Health Research Global Surgery Unit to create awareness for the study and mobilise surgeons from every state across the country. The Collaborative also informed the Federal Ministry of Health of the commencement of the study. Eligible hospitals were public or private institutions routinely performing abdominal operations at the secondary or tertiary level. A lead surgeon in each hospital constituted a team of at least three surgeons for the study and indicated interest to the steering committee.

All children and adults undergoing emergency or elective abdominal operations for benign, malignant and traumatic conditions were eligible for recruitment. Operations with anticipated clean-contaminated, contaminated or dirty surgical wounds and an abdominal incision of at least 3 cm in children or 5 cm in adults carried out by either an open or laparoscopic approach (if extraction site meets the criteria) were included. All caesarean sections were excluded. Participants considered unable to complete follow-up at postoperative day 30 and those already enrolled in another trial assessing SSI were excluded.

### Ethical statement

A dedicated RedCap platform hosted by the Nigeria hub of the NIHR Global Surgery Unit was utilised for data collection. The study was also publicly registered (ISCTRN 14422305). All adult patients were to provide written consent before recruitment. While parents or legal guardians provided consent for paediatric participants, assent was also obtained from those aged 7 years or older. Participants who were unable or unwilling to consent were excluded from the study.

### Patient and public involvement

The Association of Surgeons in Nigeria, at the inception of this research, wrote to the Nigerian Federal Ministry of Health informing them of the design of this study. A public-facing meeting was then organised in Lagos, where feedback was collated. The publication of this manuscript will further support its dissemination throughout Nigeria and across sub-Saharan Africa.

### Protocol

Each recruited patient was assigned a study number and a case report form for data collection. In this was recorded patients’ characteristics including date of birth, sex, smoking status, presence and control status of comorbidities (hypertension and diabetes), and the existence of immunosuppression with Human Immuno-deficiency Virus (HIV) and or prolonged use of steroids. Patients’ HIV status was classified as known positive, known negative and status unknown. The date of operation, indication for operation, timing of operation as elective or emergency and preoperative packed cell volume (PCV) were obtained. We explored the surgical culture in each hospital regarding recommendations to reduce SSIs thereby obtaining data for each patient on preoperative bathing with soap and water, use of the WHO Surgical Safety Checklist, type of antiseptic used for skin preparation as well as routine change of gloves and instruments for abdominal wound closure.

We documented details of the operations performed, including the date of operation, type and time of hair removal at the site of the proposed incision, American Society of Anaesthesiologists (ASA) grade, and type of anaesthesia used, cadre of lead surgeon, intraoperative pulse oximetry, prophylactic antibiotics, antiseptic used for skin preparation, operative approach including open or laparoscopic technique and actual intraoperative contamination. We defined a wound as clean when it is surgically created with no inflammation encountered during the procedure, with no break in aseptic technique, and during which the alimentary, respiratory and genitourinary tracts are not entered. A clean-contaminated wound is an incision carried out on the respiratory, alimentary or genitourinary tract without contamination from luminal contents. A contaminated wound is one in which there is a major break in aseptic technique, gross spillage from the gastrointestinal tract or operation on an inflamed area of the body. A dirty wound is one in which pus is encountered during the operation or viscera are perforated, including traumatic wounds with delayed presentation or devitalised tissue. Other variables recorded include intraoperative blood sugar monitoring, intraoperative temperature monitoring, final operation performed, skin closure technique and total duration of operation in minutes.

### Study outcome

All patients were evaluated at discharge and on postoperative day 30 for the study outcomes. Our primary outcome measure was the incidence of SSI within postoperative day 30. We adopted the US Centre for Disease Control and Prevention criteria for the detection and grading of SSI.^1 11^ A patient was adjudged to have superficial SSI with at least one of the following: (1) purulent drainage from the superficial or deep; (2) pain or tenderness, localised swelling, redness, heat or fever, or several of these symptoms, and the incision is opened deliberately or spontaneously dehisces or (3) clinically or radiologically detected abscess within the wound. Organ space infections were recorded separately and defined as intra-abdominal or pelvic infections detected clinically or symptomatically, radiologically or intraoperatively. A mandatory online training was completed by all collaborators before the commencement of data collection.

The secondary objectives were to determine the influence of SSI on postoperative length of stay, readmission, reoperation or mortality within 30 days of abdominal operations. Patients who died during hospitalisation were excluded only if death occurred before a SSI could be assessed and was unrelated to infection. Postoperative length of stay was calculated from the date of operation to the date of discharge (or death, in instances of mortality). In all instances, follow-up was carried out in person.

This manuscript was prepared in adherence to STrengthening the Reporting of Observational studies in Epidemiology (STROBE) guidelines for observational reporting.^12^

### Sample size

The study was designed to estimate an SSI rate of approximately 20% as observed in the CHEETAH trial,^13^ with a 95% confidence level. A total of 50 hospitals with an average of 40 patients per cluster (total *n*=2000) were planned. Accounting for an intraclass correlation coefficient of 0.01 observed in our previous global studies,^14^ the design effect was calculated as 1.39, resulting in an effective sample size of approximately 1439 patients. This yields a margin of error of 2.1%, indicating that the 95% CI for the estimated proportion would be approximately 0.18–0.22. Assuming a 10% loss to follow-up or death before SSI diagnosis, the minimum sample size required is approximately 2220.

### Statistical analyses

Categorical demographic and intraoperative variables were summarised using frequencies and percentages and continuous variables using means with SD. Medians and IQRs were also calculated for skewed continuous variables. Analyses of secondary outcomes were exploratory and aimed to identify associations rather than causal relationships. Univariate logistic regressions were used to assess the unadjusted association between variables and outcomes, accounting for clustering of hospitals. A multilevel logistic regression model was fitted to explore factors associated with SSI at 30 days postoperatively. Predefined variables (age, preoperative PCV, urgency of surgery, ASA grade, contamination, operative approach, WHO checklist use, type of anaesthesia preoperative bath and change of gloves and instruments before wound closure) were included in the model. Hospital was included in the model as a random intercept to account for clustering of patients within hospitals using maximum likelihood method with adaptive quadrature. Key model assumptions were validated, and multicollinearity issues were tested using the variance inflation factor. Missing data were assessed for each variable, and complete-case analysis was performed. Continuous variables were examined for distributional assumptions and retained as continuous predictors in the regression models. The resulting ORs and 95% CIs are presented with p values. All statistical analyses were performed using Stata (StataCorp. 2023. Stata Statistical Software: Release V.17. College Station, TX: StataCorp LLC) and R V.4.4.2 (R Foundation for Statistical Computing, Vienna, Austria; Data Supplement).

## Results

**Figure 1:**
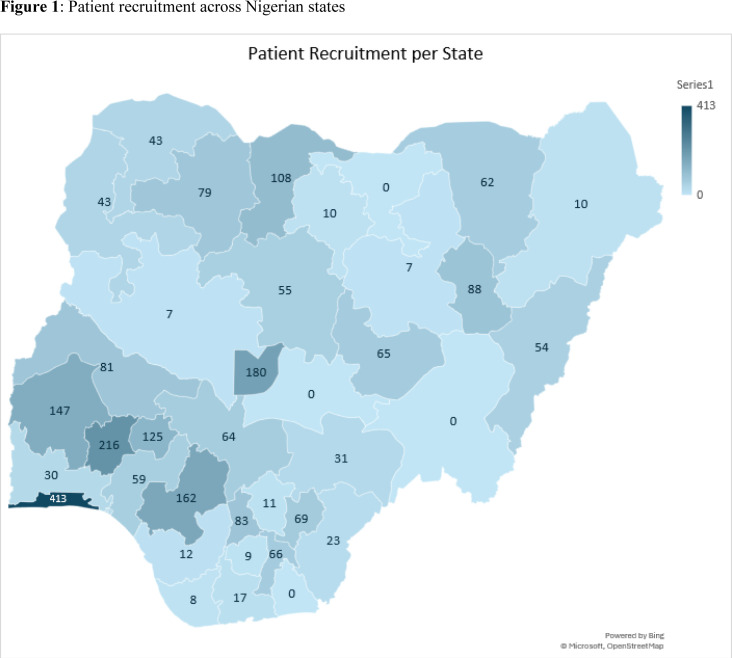
Patient recruitment across Nigerian states.

Seventy-two hospitals signified their intention to join this collaborative research, out of which 56 hospitals obtained local institutional approval and were given access to patient recruitment pages on our dedicated REDCap platform. Overall, 53 hospitals recruited 2466 patients over 4 months. Patients were recruited from the Federal Capital Territory and 32 of the 36 states of Nigeria. We could not identify any committed hospital lead in Jigawa, Taraba, Nassarawa and Akwa Ibom states before the closure of recruitment. The recruitment across the country is as depicted in figure 1. The hospitals include 35 federal government-funded hospitals, 13 state government-funded hospitals, three missions and two private hospitals. Of these, 84.9% (45 of 53) were tertiary hospitals, while the rest offered secondary-level care. The hospital leads include senior professors, consultants and trainees who constituted a local team of at least three surgeons. Most institutions recruited across different surgical specialties including general surgery, paediatric surgery and urology. Recruitments per hospital is depicted in online supplemental figure 1.

**Figure 2:**
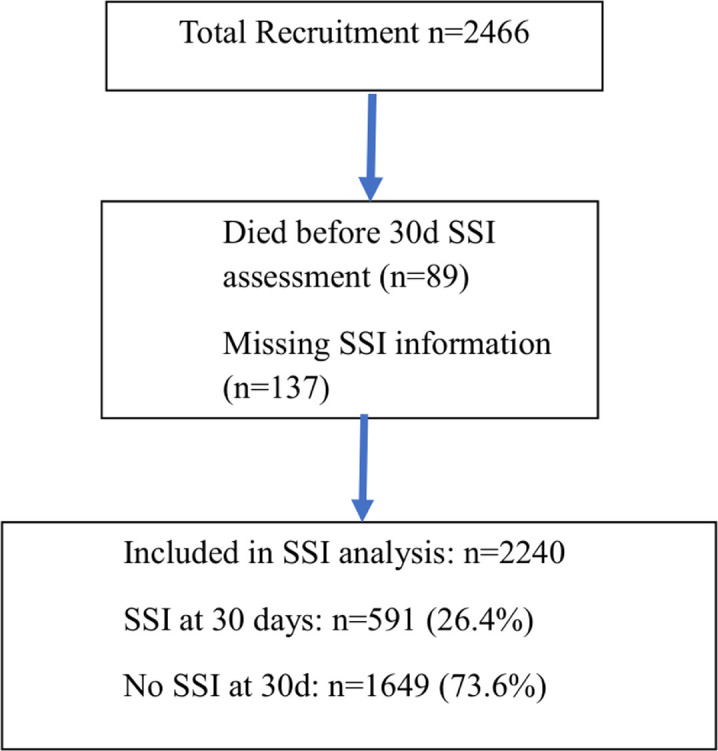
Flowchart of patients included in the study. SSI, surgical site infection.

Of the total 2466 patients recruited, 137 had missing information on SSI including 135 who died on admission along with two others with incomplete records. These were excluded from SSI analysis since the primary outcome measures of the study could not be determined. Following discharge, another 89 patients died before SSI assessment were excluded; all deaths were reviewed and confirmed to be unrelated to SSI. A total of 2240 patients were subsequently included in the final SSI analysis, of which 591 (26.4%) had SSI, as depicted in figure 2. The majority of the SSI, 482 of 591 (81.6%), were diagnosed before or at the time of discharge.

The baseline sociodemographic characteristics of patients are depicted in table 1. Males and females were nearly equal in distribution among the patients, while the age ranged from 2 days to 95 years, with a mean of 31.4 years (SD=21.5), and a median age of 30 years (IQR 13 to 46). Mean preoperative PCV was 34.7 (SD=6.2) with a median 34.2 (IQR 31.0 to 38.0). Only 85 patients (3.8%) were previously diagnosed with diabetes mellitus, while a larger proportion (12.8%) are known hypertensive patients. Preoperative diagnosis of HIV was made in 29 (1.3%), 73 (3.3%) had been on chronic use of steroids, while 483 (21.6%) were not aware of their HIV status before the operation.

**Table 1:** Baseline demographics by SSI status

	No SSI (n=1649)	SSI (n=591)	Total (n=2240)	P value
Gender, n (%)				
Female	798 (48.4)	334 (56.5)	1132 (50.6)	0.048
Male	850 (51.6)	257 (43.5)	1107 (49.4)	
Missing	1	0	1	
Age (years)				
Mean (SD)	32.1 (21.1)	29.5 (22.5)	31.4 (21.5)	0.745
Median (IQR)	31 (14, 46)	27 (10, 46)	30 (13, 46)	0.125
Missing	16	13	29	
ASA grade, n(%)				
1–2	1218 (74.6)	308 (52.5)	1526 (68.8)	<0.001
3–5	414 (25.4)	279 (47.5)	693 (31.2)	
Missing	17	4	21	
Preoperative PCV (%)				
Mean (SD)	35.0 (6.2)	33.8 (6.2)	34.7 (6.2)	<0.001
Median (IQR)	35.0 (31.2 to 38.0)	33.0 (30.0 to 37.0)	34.2 (31.0 to 38.0)	<0.001
Missing	25	12	37	
Diabetes, n (%)				
No	1583 (96.3)	566 (95.8)	2149 (96.2)	0.151
Yes	60 (3.7)	25 (4.2)	85 (3.8)	
Missing	6	0	6	
Hypertension, n (%)				
No	1427 (86.8)	522 (88.3)	1949 (87.2)	0.445
Yes	217 (13.2)	69 (11.7)	286 (12.8)	
Missing	5	0	5	
HIV status, n (%)				
Negative	1292 (78.4)	435 (73.6)	1727 (77.1)	0.524
Positive	22 (1.3)	7 (1.2)	29 (1.3)	
Not known	334 (20.3)	149 (25.2)	483 (21.6)	
Missing	1	0	1	
Current smoker, n (%)				
No	1620 (98.7)	570 (96.4)	2190 (98.1)	0.030
Yes	22 (1.3)	21 (3.6)	43 (1.9)	
Missing	7	0	7	
Preoperative steroid use, n (%)				
No	1597 (97.0)	567 (96.1)	2164 (96.7)	0.271
Yes	50 (3.0)	23 (3.9)	73 (3.3)	
Missing	2	1	3	
Urgency, n (%)				
Elective	800 (48.5)	169 (28.6)	969 (43.3)	<0.001
Emergency	849 (51.5)	422 (71.4)	1271 (56.7)	
Missing	0	0	0	
Preoperative bathing with soap and water, n (%)				
No	1073 (65.1)	478 (80.9)	1551 (69.2)	<0.001
Yes	576 (34.9)	113 (19.1)	689 (30.8)	
Missing	0	0	0	

ASA, American Society of Anaesthesiologists; PCV, packed cell volume; SSI, surgical site infection.

The majority of operations (1355, 60.5%) were carried out by consultant surgeons, senior registrars 717 (32%), registrars 95 (4.2%), senior medical officers 33 (1.5%) and medical officers 40 (1.8%). Procedures were wide-ranging as shown in online supplemental table 1. They include colon and rectal 336, gynaecology 334, small bowel operations 247, urological procedures 259, hernia 109, oesophagus and stomach 72 and a mix of general surgery 880 (including appendectomies, exploratory laparotomy for perforated viscus). The majority (1864, 83.2%) of operations included were for benign conditions, 304 (13.6%) were for malignancies and 71 (3.2%) were for trauma. Regarding timing, 969 (43.3%) operations were carried out in planned elective settings while more than half, 1271 (56.7%), were emergency operations. We limited recruitment to operations with planned abdominal incisions greater than 3 cm in children and 5 cm in adults; nearly all cases (2191, 97.9%) had open operations, while 41 (1.8%) had laparoscopic operations with 6 (0.3%) cases converted to open laparotomy. Of those who had open operations, 1219 (54.4%) had midline incisions, hair removal was not performed in 918 (41.0%) of patients and was carried out with a razor before arrival in the theatre in 762 (34.0%) cases, with a razor in the theatre in 641 (28.6%) cases and in theatre with electric clipper in 27 (1.2%) patients (table 2).

**Table 2:** Intraoperative details and postoperative outcomes by SSI status

	No SSI (n=1649)	SSI (n=591)	Total (n=2240)	P value
WHO surgical checklist, n (%)				
No	470 (28.5)	205 (34.7)	675 (30.1)	0.028
Yes	1178 (71.5)	386 (65.3)	1564 (69.9)	
Missing	1	0	1	
Actual contamination, n (%)				
Clean	249 (15.1)	28 (4.7)	277 (11.4)	<0.001
Clean-contaminated	970 (58.8)	192 (32.5)	1162 (51.3)	
Contaminated	266 (16.1)	157 (26.6)	423 (20.1)	
Dirty	164 (9.9)	214 (36.2)	378 (17.1)	
Missing	0	0	0	
Operative approach, n (%)				
Laparoscopic	37 (2.2)	4 (0.7)	41 (1.8)	<0.001
Laparoscopic converted to open	4 (0.2)	2 (0.3)	6 (0.3)	
Open-midline	815 (49.4)	397 (67.4)	1212 (54.2)	
Open-other incisions	793 (48.1)	186 (31.6)	979 (43.7)	
Missing	0	2	2	
Indication for operation, n (%)				
Benign	1373 (83.3)	491 (83.2)	1864 (83.3)	0.771
Malignant	231 (14.0)	73 (12.4)	304 (13.6)	
Trauma	45 (2.7)	26 (4.4)	71 (3.2)	
Missing	0	1	1	
Anaesthesia, n (%)				
General	1222 (74.2)	519 (88.0)	1741 (77.8)	<0.001
Regional	426 (25.8)	71 (12.0)	497 (22.2)	
Missing	1	1	2	
Intraoperative pulse oximetry, n (%)				
No	43 (2.6)	7 (1.2)	50 (2.2)	0.027
Yes	1606 (97.4)	583 (98.8)	2189 (97.8)	
Missing	0	1	1	
Prophylactic antibiotics given, n (%)				
No	53 (3.2)	15 (2.5)	68 (3.0)	0.198
Yes	1594 (96.8)	576 (97.5)	2170 (97.0)	
Missing	2	0	2	
Hair removal at site of wound, n (%)				
No	595 (36.1)	319 (54.0)	914 (40.8)	<0.001
Yes	1053 (63.9)	272 (46.0)	1325 (59.2)	
Missing	1	0	1	
Skin closure, n (%)				
Clips	448 (27.2)	110 (18.6)	558 (24.9)	<0.001
Interrupted suture	744 (45.2)	412 (69.7)	1156 (51.7)	
Subcuticular suture	449 (27.3)	64 (10.8)	513 (22.9)	
Skin left open	6 (0.4)	5 (0.8)	11 (0.5)	
Missing	2	0	2	
Death at 30 days				
No	1625 (99.1)	523 (89.9)	2148 (96.7)	<0.001
Yes	15 (0.9)	59 (10.1)	74 (3.3)	
Readmission at 30 days				
No	1631 (99.0)	497 (92.7)	2128 (97.4)	<0.001
Yes	17 (1.0)	39 (7.3)	56 (2.6)	
Reoperation at 30 days				
No	1640 (99.5)	504 (93.7)	2144 (98.0)	<0.001
Yes	9 (0.5)	34 (6.3)	43 (2.0)	
Length of stay (days)				
Median (IQR)	8 (5, 12)	14 (9, 22)	9 (6, 15)	<0.001

SSI, surgical site infection.

General anaesthesia was employed in 1741 (77.8%) operations, while regional anaesthesia was adopted in 497 (22.2%) operations. Nearly all patients, 2170 (97%), had prophylactic antibiotics administered before induction of anaesthesia, and 2189 (97.8%) were monitored with pulse oximeters intraoperatively. The WHO Surgical Safety Checklist was utilised in 1564 (69.9%) cases but not in 675 (30.1%). On completion of the procedure, abdominal wounds were closed with interrupted sutures in 1156 (51.7%) cases, with subcuticular sutures in 513 (22.9%), skin staples in 558 (24.9%) and the skin was left open in 11 (0.5%) cases.

Overall, 591 patients (26.4%) had SSI within 30 days of operation. Preoperative characteristics influencing SSI are as shown in table 1. There were statistically significant associations between the occurrence of SSI and preoperative PCV (p<0.001), current smoking status (p=0.03), emergency operations (p<0.001), ASA grades 3–5 (p<0.001) and preoperative bathing with soap and water (p<0.001). The age of patients, presence of comorbidities such as hypertension or diabetes mellitus and chronic use of steroids were not significantly associated with the occurrence of SSI in this cohort. Intraoperative factors associated with the occurrence of SSI (table 2) include actual intraoperative contamination (p<0.001), operative approach adopted (p<0.001), technique of anaesthesia employed (p<0.001), hair removal method used (p<0.001), method of skin closure (p<0.001) and routine change of gloves by the entire surgical team (p<0.001).

In a multivariable analysis (table 3), ASA grades 3–5: OR 1.91 (1.48 to 2.45, p<0.001), contaminated/dirty contamination: OR 5.48 (3.33 to 9.01, p<0.001) and open midline approach: OR 1.41 (1.06 to 1.88, p=0.020) were significantly associated with higher odds of SSI. Adoption of the culture of change of gloves by all team members before skin closure, change of instruments or both was only carried out in 10.3%–16.9% of operations (online supplemental table 2). This however reduced the proportion of patients developing SSI independently and across hospitals with different SSI rates. We also investigated the influence of adoption of intraoperative measures such as antiseptic solutions employed for preoperative skin preparation and change of gloves or instruments before abdominal wall closure among hospitals with low (<20%) SSI rates versus those with medium (20%–40%) or high (>40%) rates as shown in online supplemental table 3. No trend in the adoption of measures was observed among the hospital categories.

**Table 3:** Multivariable logistic regression analysis of factors associated with SSI

Factor	OR (95% CI)	P value
Age	1.00 (0.99 to 1.01)	0.615
Preoperative PCV	0.98 (0.97 to 1.00)	0.095
Urgency		
Elective	Reference*	
Emergency	1.09 (0.82 to 1.44)	0.542
ASA grade		
Grades 1–2	Reference*	
Grades 3–5	1.91 (1.48 to 2.45)	<0.001
Actual contamination		
Clean	Reference*	
Clean-contaminated	1.78 (1.11 to 2.87)	0.017
Contaminated/dirty	5.48 (3.33 to 9.01)	<0.001
Operative approach		
Open non-midline	Reference*	
Open-midline	1.41 (1.06 to 1.88)	0.020
Laparoscopic	1.25 (0.47 to 3.31)	0.652
Use of WHO checklist		
No	Reference*	
Yes	1.10 (0.80 to 1.49)	0.564
Anaesthesia		
General	Reference*	
Regional	0.76 (0.53 to 1.09)	0.142
Preoperative bath		
No	Reference*	
Yes	0.78 (0.56 to 1.08)	0.133
Change of gloves and instruments before closure		
No	Reference*	
Yes	1.22 (0.80 to 1.84)	0.357

* Results are from a multilevel logistic regression model adjusted for predefined variables (age, preoperative packed cell volume, urgency of surgery, ASA grade, contamination, operative approach, WHO checklist use, type of anaesthesia preoperative bath and change of gloves and instruments before wound closure). Hospital was included in the model as random intercept to account for clustering of patients within hospitals. Estimated variance of the hospital-level random intercept=0.584 (SE 0.187), intraclass correlation coefficient=0.151 (SE 0.041).

ASA, American Society of Anaesthesiologists; PCV, packed cell volume; SSI, surgical site infection.

At the 30-day follow-up, 56 patients (2.5%) had been readmitted after discharge. Among them, 43 (2.0%) required reoperation, with 23 cases (1.1%) attributed to SSI. In total, 74 patients (3.3%) died within 30 days of undergoing abdominal surgery, and 59 of these deaths (79.7%) were associated with SSI. Patients who developed SSI had a longer postoperative hospital stay, with a median of 14 days (IQR 9–22), compared with 8 days (IQR 5–12) for those without SSI (p<0.001; see table 2).

## Discussion

Registries tracking surgical procedures and outcomes require significant financial and logistical resources, making them impractical in many LMICs. Snapshot audits of clinical practice offer an alternative, providing valuable data to guide policy decisions, monitor adherence to best practices, and assess patient outcomes.

This study marks the first nationwide, prospective, multicentre observational study in Nigeria aimed at evaluating SSI incidence following abdominal operations. It represents a milestone for the Nigerian surgical community, demonstrating the power of national surgical collaboration, with participation from 53 hospitals across the country. The study also highlights the strong research potential among Nigerian surgeons, encompassing secondary and tertiary hospitals, publicly funded state and federal-owned facilities, private institutions and mission hospitals. The findings offer critical evidence for shaping policies on SSI prevention in Nigeria and similar sub-Saharan African settings.

The study identified an SSI rate of 26.4%, which was significantly associated with extended hospital stays, increased rates of readmission and reoperation and elevated 30-day postoperative mortality. In comparison, a previous multinational analysis of abdominal surgeries conducted across 66 countries reported a global SSI rate of 12.3%.^14^ This rate varied by HDI: 9.4% in high-HDI countries, 14.0% in middle-HDI countries and 23.2% in low-HDI countries. Nakhleh *et al*^15^ further demonstrated that the economic burden of SSIs is disproportionately higher in sub-Saharan Africa compared with Western regions. The elevated SSI rate observed in Nigeria underscores a significant public health challenge that warrants immediate attention and targeted intervention.

A meta-analysis of 78 studies involving 38 187 patients who underwent emergency surgery in sub-Saharan Africa found a SSI rate of 14.4%.^16^ Most of these studies were retrospective, and only 12.8% were rated as high quality by the authors. In contrast, our prospective study observed that emergency surgeries—making up the majority of cases—had double the SSI risk compared with elective procedures (33.2% vs 17.4%). ASA grades were significantly associated with SSI risk, with nearly one-third of patients classified as ASA grades 3–5, facing double the SSI risk compared with ASA grades 1–2 (40.3% vs 20.2%). Additionally, over one-third of procedures involved contaminated or dirty wounds, with SSI rates of 37.1% and 56.6%, respectively, compared with 16.5% in clean-contaminated cases. Multiple risk prediction models have been developed to estimate the likelihood of SSIs following abdominal procedures.^17 18^ These models will be incorporated into future intervention strategies to enhance patient outcomes and guide clinical decision-making

The current study highlights a critical link between postoperative SSI and adverse outcomes such as elevated rates of hospital readmission, reoperation and 30-day mortality following abdominal surgeries. These findings underscore the serious clinical and economic implications of SSI, particularly in LMICs, where similar research has consistently revealed a substantial burden.^1319^,^22^ The convergence of evidence across diverse settings emphasises the urgent need for targeted interventions, improved perioperative care and robust infection prevention strategies to mitigate the impact of SSI and enhance surgical outcomes globally.

The WHO Surgical Safety Checklist was widely used intraoperatively and correlated with lower SSI rates. However, 30% of cases did not use the checklist, necessitating further studies to identify barriers and improve compliance. Conversely, routine glove changes by all scrubbed team members before wound closure and the use of clean instruments for closure^14^—policies endorsed by the Nigerian Federal Ministry of Health and communicated to all Federal Teaching Hospitals—were rarely practised. Expanding educational initiatives across public and private surgical facilities may improve adherence and further reduce SSI rates nationwide. Other potentially modifiable preoperative factors influencing SSI identified, such as low preoperative haemoglobin, smoking and routine preoperative shower, can be subsequently tested in elective patients on a national scale to reduce SSI.

### Strengths of the study

This study has established real-time baseline data for surgical patients across Nigeria, providing valuable insights for national adoption. The truly national collaboration, encompassing representation from every geopolitical and social stratum, has sparked widespread enthusiasm among surgeons for data-driven surgical interventions and improved patient outcomes.

The engagement of the Federal Ministry of Health by this collaborative effort is expected to shape policy reforms, drive practice improvements and support the implementation of proven interventions for SSI reduction. Another key strength of this study is its comprehensive inclusion of emergency operations, reflecting the diverse range of abdominal procedures performed nationwide.

### Limitations

Our study focused exclusively on current practices in collaborating hospitals. As a result, we did not gather sufficient data on bacterial culture and antimicrobial resistance patterns in infected wounds. This remains an important area of interest for our research group and will be incorporated into future studies. Additionally, we did not collect data on the cost of treating SSI, which would have been valuable to healthcare policymakers. However, due to the complexity of hospital funding structures—which vary significantly across public, private and mission hospitals—obtaining comprehensive cost data would have presented a considerable challenge. The heterogeneity of cases included, local hospital cultures and individual nuances are confounders that were not sufficiently tested in this study and will be incorporated into our future studies.

### Prospects

Several studies have reported improvement of surgical outcomes in terms of SSI and mortality with increased surveillance.^22 23^ The findings of this study provide the basis for national planning, funding and conduct of high-quality surgical research aimed at reducing SSI. In conjunction with the Federal and State ministries of health, we will design randomised trials on SSI prevention, targeting specific preoperative and intraoperative risk factors we have identified. Educational interventions will also be provided for surgeons and surgical teams to improve the adoption of preventive measures. The success of this collaborative study is a motivation for its expansion. We will utilise the strength of the group to carry out more outcome assessments in surgical practice in the country

## Conclusion

Abdominal surgeries in Nigeria are associated with a high SSI rate, significantly increasing the risks of readmission, reoperation and mortality within 30 days. These adverse outcomes are largely driven by operations on critically ill patients. Strengthening the adoption of preventive measures has the potential to significantly reduce this burden, improving patient outcomes and alleviating the strain on both individuals and the healthcare system. Targeted interventions will be essential in minimising SSI rates and promoting safer surgical practices nationwide.

## Supplementary material

10.1136/bmjgh-2025-021423 online supplemental file 2

## Data Availability

Data available on RedCap upon reasonable request.
