# Supplementary material for: Surgical site infections after abdominal surgeries: a prospective multicentre study in 53 Nigerian hospitals
Source: BMJ Glob Health. 2026 Jun 4;11(6):e021423. doi: 10.1136/bmjgh-2025-021423 (PMC13239510; doi:10.1136/bmjgh-2025-021423)

SUPPLEMENTARY TABLES AND FIGURES

**Supplementary Table 1: Operative details of included patients:**

| Procedures | No SSI  (n=1649) | SSI  (n=591) | Total  (n=2240) |
| --- | --- | --- | --- |
| COLON & RECTUM | 212 (12.9%) | 101 (17.2%) | 313 (14.0%) |
| GALLBLADDER & BILIARY TREE | 29 (1.8%) | 9 (1.5%) | 38 (1.7%) |
| GENERAL SURGERY | 559 (33.9%) | 191 (32.5%) | 750 (33.6%) |
| GYNAECOLOGY | 295 (17.9%) | 35 (6.0%) | 330 (14.8%) |
| HERNIA | 86 (5.2%) | 25 (4.3%) | 111 (5.0%) |
| LIVER | 2 (0.1%) | 2 (0.3%) | 4 (0.2%) |
| OESOPHAGUS & STOMACH | 41 (2.5%) | 26 (4.4%) | 67 (3.0%) |
| PANCREAS | 6 (0.4%) | 2 (0.3%) | 8 (0.4%) |
| SARCOMA | 1 (0.1%) | 1 (0.2%) | 2 (0.1%) |
| SMALL BOWEL | 102 (6.2%) | 134 (22.8%) | 236 (10.6%) |
| SPLEEN | 17 (1.0%) | 2 (0.3%) | 19 (0.9%) |
| UROLOGY | 231 (14.0%) | 29 (4.9%) | 260 (11.6%) |
| OTHER | 65 (3.9%) | 31 (5.3%) | 96 (4.3%) |
| MISSING | 3 | 3 | 6 |

**Supplementary Table 2: Prevention measures by SSI status**

|  | No SSI  (n=1649) | SSI  (n=591) | Total  (n=2240) | P-value |
| --- | --- | --- | --- | --- |
| Savlon, n(%) | | | | |
| No | 190 (11.5) | 77 (13.0) | 267 (11.9) | 0.015 |
| Yes | 1459 (88.5) | 514 (87.0) | 1973 (88.1) |  |
| Missing | 0 | 0 | 0 |  |
| Methylated spirit, n(%) | | | | |
| No | 766 (46.5) | 223 (37.7) | 989 (44.2) | 0.018 |
| Yes | 883 (53.5) | 368 (62.3) | 1251 (55.8) |  |
| Missing | 0 | 0 | 0 |  |
| Chlorhexidine, n(%) | | | | |
| No | 1593 (96.6) | 564 (95.4) | 2157 (96.3) | 0.146 |
| Yes | 56 (3.4) | 27 (4.6) | 83 (3.7) |  |
| Missing | 0 | 0 | 0 |  |
| Povidone iodine, n(%) | | | | |
| No | 578 (35.1) | 222 (37.6) | 800 (35.7) | 0.963 |
| Yes | 1071 (64.9) | 369 (62.4) | 1440 (64.3) |  |
| Missing | 0 | 0 | 0 |  |
| Change of instruments for closure, n(%) | | | | |
| No | 1391 (84.4) | 470 (79.5) | 1861 (83.1) | 0.080 |
| Yes | 257 (15.6) | 121 (20.5) | 378 (16.9) |  |
| Missing | 1 | 0 | 1 |  |
| Change of gloves before skin closure, n(%) | | | | |
| No | 1419 (86.2) | 460 (77.8) | 1879 (84.0) | <0.001 |
| Yes | 227 (13.8) | 131 (22.2) | 358 (16.0) |  |
| Missing | 3 | 0 | 3 |  |
| Change of gloves and instruments before closure, n(%) | | | | |
| No | 1496 (90.9) | 510 (86.3) | 2007 (89.7) | 0.075 |
| Yes | 150 (9.1) | 81 (13.7) | 231 (10.3) |  |
| Missing | 2 | 0 | 2 |  |

**Supplementary Table 3: Prevention measures by hospital SSI rate category**

|  | Hospital SSI rate <20%  (n=1006) | Hospital SSI rate 20% - 40%  (n=1033) | Hospital SSI rate >40%  (n=427) |
| --- | --- | --- | --- |
| Savlon, n(%) | | | |
| No | 107 (10.6%) | 155 (15.0%) | 28 (6.6%) |
| Yes | 899 (89.4%) | 878 (85.0%) | 399 (93.4%) |
| Missing | 0 | 0 | 0 |
| Methylated spirit, n(%) | | | |
| No | 584 (58.1%) | 295 (28.6%) | 212 (49.6%) |
| Yes | 422 (41.9%) | 738 (71.4%) | 215 (50.4%) |
| Missing | 0 | 0 | 0 |
| Chlorhexidine, n(%) | | | |
| No | 962 (95.6%) | 999 (96.7%) | 417 (97.7%) |
| Yes | 44 (4.4%) | 34 (3.3%) | 10 (2.3%) |
| Missing | 0 | 0 | 0 |
| Povidone iodine, n(%) | | | |
| No | 288 (28.6%) | 463 (44.8%) | 122 (28.6%) |
| Yes | 718 (71.4%) | 570 (55.2%) | 305 (71.4%) |
| Missing | 0 | 0 | 0 |
| Change of instruments for closure, n(%) | | | |
| No | 862 (85.9%) | 836 (81.1%) | 355 (83.1%) |
| Yes | 142 (14.1%) | 195 (18.9%) | 72 (16.9%) |
| Missing | 2 | 2 | 0 |
| Change of gloves before skin closure, n(%) | | | |
| No | 882 (87.8%) | 837 (81.3%) | 347 (81.3%) |
| Yes | 122 (12.2%) | 192 (18.7%) | 80 (18.7%) |
| Missing | 2 | 4 | 0 |
| Change of gloves and instruments before closure, n(%) | | | |
| No | 932 (92.8%) | 907 (88.1%) | 375 (87.8%) |
| Yes | 72 ( 7.2%) | 123 (11.9%) | 52 (12.2%) |
| Missing | 2 | 3 | 0 |

Supplementary Figure 1: Patient recruitment per hospital


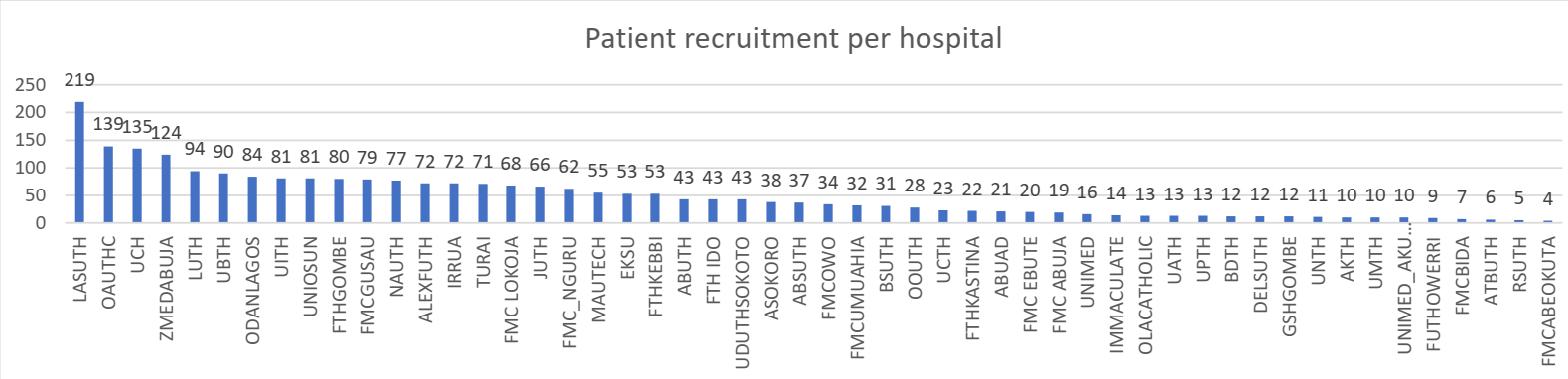

Supplement: online supplemental file 2 [file bmjgh-11-6-s001.docx]
